# Comparative Analysis of Various Rotator Cuff Stretching Techniques: Efficacy and Recommendations for Gym Enthusiasts

**DOI:** 10.7759/cureus.51785

**Published:** 2024-01-07

**Authors:** Prajyot Ankar, Pallavi Harjpal

**Affiliations:** 1 Neurophysiotherapy, Ravi Nair Physiotherapy College, Datta Meghe Institute of Higher Education and Research, Wardha, IND

**Keywords:** systematic review, rotator cuff health, shoulder flexibility, range of motion, stretching, physiotherapy, gym enthusiasts, rotator cuff stretching

## Abstract

Shoulder pain is a common complaint among gym-going individuals, particularly those engaged in upper limb workouts. The rotator cuff, comprising four muscles, plays a crucial role in stabilizing the shoulder joint during movements and supporting its mobility. Imbalances or weaknesses in these muscles can lead to shoulder injuries, affecting performance and overall well-being. The main aim of this review is to explore the benefit of one of the approaches in preventing shoulder pain and improving performance among gym-going individuals. Specific rotator cuff stretching exercises target the entire shoulder complex to enhance the mobility, control, and stabilization of the joint. The dynamic warm-up routine will actively engage the relevant muscles in various planes of motion, promoting the increased range of motion and reduced inflammation. Ultimately, the results from this review can serve as important knowledge for gym-going individuals, trainers, and fitness enthusiasts, guiding them in incorporating evidence-based warm-up strategies to optimize their workouts. Empowering individuals to take proactive measures in caring for their shoulder health can lead to improved overall performance and a better training experience in the gym.

## Introduction and background

Four muscles make up the rotator cuff at the shoulder joint. In the world of physical fitness, a robust and flexible shoulder complex is of paramount importance, as it underpins a myriad of upper-body movements [1]. The rotator cuff, a collection of muscles and tendons surrounding the shoulder joint, plays a pivotal role in maintaining stability and enabling a wide range of motions. This muscle increases stability of the shoulder complex. Abduction for 30 degrees is done by the muscle supraspinatus. The infraspinatus works as an external rotator. Additionally, they all serve as reinforcements for the glenohumeral (GH) joint [2]. One of the most flexible joints in the body is the shoulder joint. The group of muscles known as the rotator cuff includes the teres minor, infraspinatus, subscapularis, and supraspinatus [3,4]. The scapular glenoid cavity holds the head of the humerus firmly via the tendons that surround the shoulder joint [5]. After a shoulder injury, dull ache-like pain in the rotator cuff is possible. This pain frequently gets worse while using the arm independently of the body [6].

The stability and mobility of the shoulder joint are crucial for everyday activities. They are greatly influenced by the rotator cuff, a complex of muscles and tendons that surround and support the shoulder joint. However, gym-going individuals may experience shoulder discomfort, which is a typical complaint. Shoulder cuff muscles are responsible for maintaining the stability of the shoulder joint during movements, and any lack of balance or weakness can lead to shoulder injuries [7]. Engaging in repetitive overhead exercises, heavy weightlifting, or improper form can put additional stress on the rotator cuff muscles, leading to overuse injuries such as tendinitis or tears. In addition, poor posture or weak shoulder blade muscles can also contribute to shoulder discomfort during exercises [8,9]. Rotator cuff stretching with a warm-up is an effective exercise to reduce and manage shoulder pain. Such stretching can target the entire rotator cuff, helping to improve mobility, control, and stabilization of the shoulder joint [10]. Dynamic exercises focus on increasing the range of motion (ROM) by actively recruiting muscles around the joint in numerous planes of motion; this strengthens weakened musculature and can help reduce inflammation [11]. As part of athletic training, flexibility is a key component for promoting secure and efficient motions in sports. The flexibility training regimen includes warm-up exercises to improve GH ROM, avoid injury, and improve performance using static stretching of the upper extremities. Static stretching, however, has been shown to be potentially harmful to performance in earlier research [12]. The posterior shoulder's flexibility can be increased by performing stretches like the cross-body and sleeper stretches. Wilk made several changes to these stretching techniques. Yet, there aren't many quantitative studies on the new, modified stretching techniques [13]. In participants with posterior shoulder tightness, a recent study demonstrated the immediate benefits of stretching and soft tissue mobilization on shoulder ROM and muscular stiffness [14]. It has been hypothesized that enhanced stretch tolerances rather than decreased stiffening of the unit of muscle-tendon are responsible for the enhancement in joint ROM found after dynamic stretching [15]. Comparatively, dynamic stretching has been shown to increase muscular strength and power by engaging the set of muscles opposite of the intended muscle group. At the same time, the limbs perform their full ROM [16].

The objectives of a pre-workout warm-up include enhancing the suppleness of muscles and tendons, stimulating blood circulation toward the limbs, elevating body temperature, and facilitating smooth and synchronized movements, all of which aid in preparing the body for physical activity [17]. The outcome of this comparative analysis is poised to contribute significantly to the existing knowledge concerning rotator cuff stretching techniques. By scrutinizing and evaluating available scientific literature from esteemed sources, this review endeavours to equip gym enthusiasts with well-founded recommendations on the most efficacious and appropriate stretching methods for integration into their exercise regimens.

Pathogenesis

The most frequent cause of rotator cuff disease is old age. It is a gradual degenerative process [18,19]. Risk factors include smoking. Patients who undertake a lot of repetitive motion, such as pitchers or swimmers, or who are in their teens to early 30s are most likely to have impingement as a source of discomfort and tendon dysfunction [20,21]. Rotator cuff injuries arise as a result of trauma. The result of an acute tear caused by macrotrauma, which is frequently seen in younger individuals, is a complete tear [22,23]. Due to insufficient healing, microtrauma causes tendon deterioration and progressive tears. Degenerative tears often affect older individuals, but acute tears frequently affect younger persons. If the tendon has enough deterioration, a full tear might occur with little effort [2,24].

## Review

Methodology

**Figure 1:**
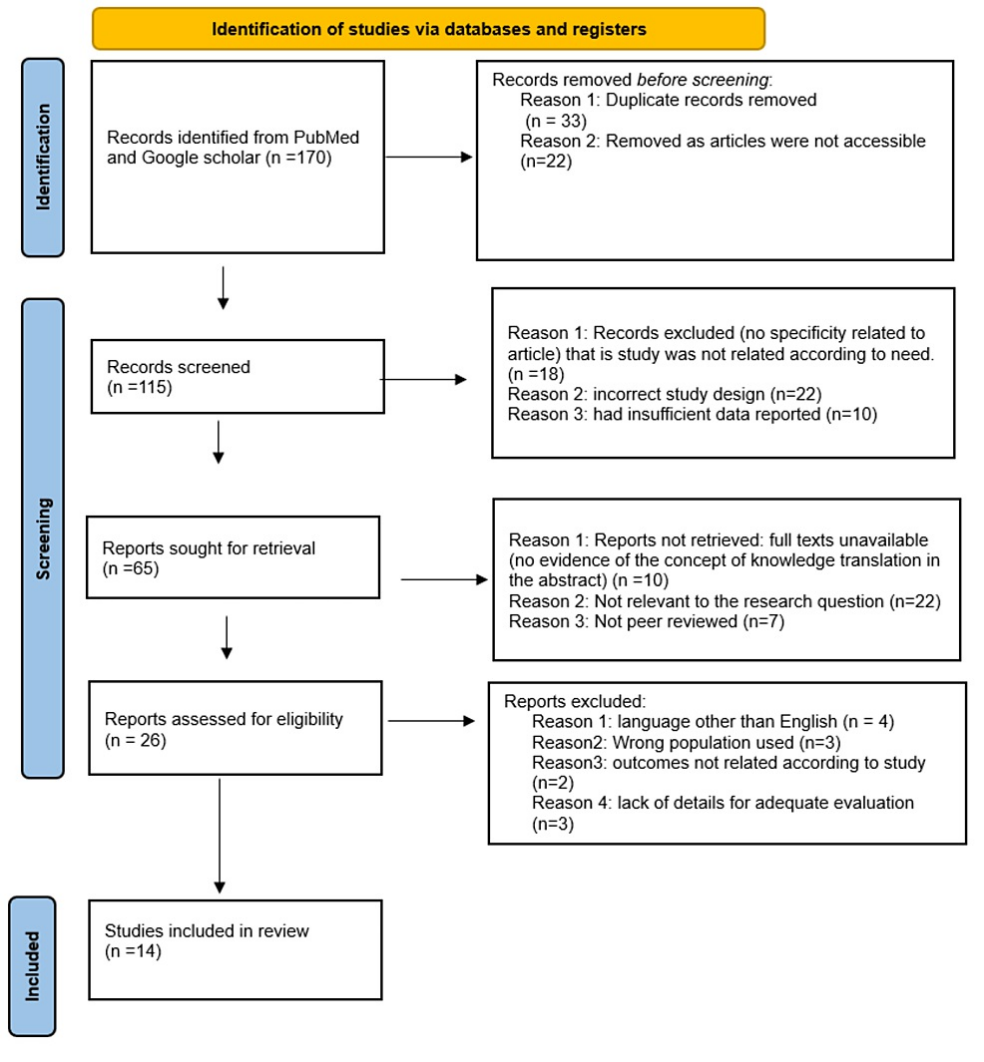
PRISMA flow diagram of the study PRISMA: Preferred Reporting Items for Systematic Reviews and Meta-Analyses

The English-language literature was searched on PubMed and Google Scholar for randomized and non-randomized clinical trials. Between 2007 and 2020, 170 articles with the keywords "rotator cuff stretching", "stretching techniques", "shoulder flexibility", "rotator cuff health", and "physiotherapy" were searched. Twelve of the 170 articles could be read in full. Studies that were included were those with patients with rotator cuff injuries along with shoulder pain, articles in the English language, and physiotherapy interventions. The exclusion criteria included any other forms of radiating pain and articles in other languages. Figure 1 shows a search of the database and data extraction. The types of articles included in this review are various randomized controlled trial, interventional studies, and review articles. There is a dearth of literature of higher levels of evidence like meta-analysis and systematic reviews.

Recent research suggests that dynamic stretching, which involves using functional motions to extend a joint's complete ROM, is a more effective warm-up method before exercise compared to static stretching [25]. While static stretching has been popular for increasing flexibility, it may actually reduce muscular strength and performance when performed before a workout [26]. On the other hand, dynamic stretching has been shown to enhance muscular function and flexibility, making it a superior choice for preparing the body for physical activity [27].

For individuals dealing with rotator cuff injuries, there is valuable research evidence supporting the inclusion of dynamic stretching as part of their treatment regimen. Ribeiro et al., in a study, provided physical therapists with a basis for treating patients with rotator cuff injuries, where participants learned to modify postures and motions that exacerbate shoulder pain. They receive a set of home exercises aimed at improving quality of life and upper limb function [28]. In the realm of sports and athletics, dynamic stretching has demonstrated numerous benefits. Çelik, in a study, highlighted the various stretching techniques, including dynamic stretching, and recommends athletes consider using static stretching after exercise or in combination with a general warm-up to avoid any negative effects on performance [29]. Table 1 shows some articles included in the review.

**Table 1:** Matrix of articles included in the review NPRS: Numerical Pain Rating Scale; ROM: range of motion; VAS: Visual Analogue Scale; WORC: Western Ontario Rotator Cuff Index; CMJ: countermovement jump; MCS: modified cross-body stretch; MSS: modified sleeper stretch; GIRD: glenohumeral internal rotation deficit; PST: posterior shoulder tightness

Sr. no.	Year	Author	Type of article	Intervention	Outcome	Intervention period	Result	Analysis
1	2020	Ribeiro et al. [28]	Randomized controlled trial	Rotator cuff loading exercise program and rotator cuff unloading exercise program	WORC, NPRS	Two times per week for 12 weeks	Unloading rotator cuff exercise program will produce better outcomes (pain reduces in unloading exercise)	Unloading exercise includes no use of weights (safe for persons with weak rotator cuff) and loading includes weights; this clearly demonstrates dynamic warm-up without any loads is more beneficial
2	2017	Çelik [29]	Randomized controlled trial	Cyclic stretching and static stretching	Flexibility: back scratch test, spike skill assessment	Two-day trial	Internal rotation strength increased in cyclic stretching group (type of dynamic stretching)	Cyclic stretching is beneficial, as it increases both the shoulder flexibility and strength
3	2016	Christensen et al. [30]	Intervention study	Theraband (Akron, Ohio, United States), against gravity with load or weights	Oxford Shoulder Score, EQ-5D-5L questionnaire, active ROM	Five months	There was deference in pain	Showed increased function in their symptomatic shoulder, reduced pain, and increased quality of life
4	2016	Corpus et al. [31]	Review article	Sleeper stretch	ROM	Four weeks	Allows posterior capsular stretching and increases ROM after stretching	Patients were found to have significant increases in total internal rotation
5	2014	Peck et al. [32]	Randomized controlled trial	Static and dynamic stretching	ROM, muscle strength		Dynamic stretching generally can be given immediately prior to activity for most athletes; this will help to prevent injury	Static stretching and passive stretching probably are reserved best for the period after activity
6	2009	McHugh and Cosgrave [33]	Review article	Static stretches	VAS	Two years	Decreases risk of injury if done after workout	Stretching makes the muscle-tendon unit more compliant. They also improve the capability to resist extreme muscle elongation and can bring down the susceptibility to a muscle strain injury
7	2008	Herman and Smith [34]	Randomized controlled trial	Static warm-up and dynamic warm-up	Performance test	Four weeks	Push-up performance was diminished in the static warm-up group after four weeks of typical static warm-up	Dynamic warm-up is more preferred prior to workouts
8	2007	McClure et al. [35]	Randomized controlled trial	Cross-body stretch, sleeper stretch	ROM	Four weeks	Internal rotation ROM improvements were substantially higher in the cross-body stretch group individuals (mean 6 SD, 20.0° 6 12.9°) than in the control group (5.9° 6 9.4°, p=0.009)	Improvement in internal rotation from the cross-body stretch was greater than for the sleeper stretch
9	2011	Samukawa et al. [16]	Randomized controlled trial	Dynamic stretching	ROM		After dynamic stretching, ankle dorsiflexion ROM was significantly higher (22.7±9.1°) than it was prior (15.4±9.6°; p<0.0001)	Findings indicate that after dynamic stretching, there were notable increase in ankle dorsiflexion ROM
10	2006	Duncan and Woodfield [36]	Randomized controlled trial	Static warm-up, dynamic warm-up	Vertical jump height scores		The scores for vertical jump (measured in meters) after no warm-up, static warm-up, and dynamic warm-up were, respectively, 0.276±0.04, 0.254±0.03, and 0.284±0.04. These results indicate that dynamic warm-up enhances children's fitness performance	When comparing the dynamic warm-up protocol to the static warm-up protocol, the vertical jump height increased significantly after dynamic warm-up
11	2016	Yamauchi et al. [13]	Randomized controlled trial	MCS, MSS	ROM	Four weeks	Shoulder internal rotation and horizontal adduction ROM were significantly increased in both groups. The MCS group experienced a decrease in teres minor muscle stiffness, while the MSS group experienced a decrease in infraspinatus muscle stiffness	The MCS and MSS are useful for reducing infraspinatus or teres minor muscle stiffness and enhancing shoulder internal rotation and horizontal adduction ROM
12	2009	Perrier et al. [37]	Randomized controlled trial	Static stretching and dynamic stretching	CMJ height	Three sessions	The average CMJ height was found to be significantly higher following dynamic stretching than following no stretching and static stretching (p=0.005 and 0.044, respectively). Compared to no stretching (41.4 cm) and static stretching (41.9 cm), dynamic stretching had a higher CMJ height (43 cm)	Dynamic stretching is more beneficial in performance (i.e., on CMJ) than static stretching
13	2022	Tawfik et al. [38]	Randomized controlled trial	Superman stretch and conventional sleeper stretch	ROM		A total of 212 shoulders were evaluated. With the exception of horizontal adduction, which only increased in the superman stretch group, both stretches showed notable increases in ROM	Comparing the superman stretch to the conventional sleeper stretch, the former may be more effective in generating rapid gains in horizontal adduction
14	2019	Jo and Kim [39]	Randomized controlled trial	Sleeper stretching and cross-body stretching techniques	ROM		Techniques for cross-body and sleeper stretching were used for 30 seconds, with three to five repetitions demonstrating beneficial effects. Furthermore, using such stretching techniques in conjunction with scapular stabilization and joint mobilization demonstrated even better results	Sleeper stretches and cross-body stretches for throwing athletes with scapular stability and joint mobilization are advised in order to avoid and cure GIRD and PST, which basically supports dynamic stretching

Christensen et al. studied that patients with non-repairable rotator cuff injuries had enhanced quality of life, decreased discomfort, and greater shoulder function after completing a five-month exercise regimen. As a result, this study encourages physiotherapists to provide exercise treatment to patients who have irreversible rotator cuff tears [30]. In the Corpus et al. study, patients were shown that they have substantial gains in total internal rotation (IR) and also a 38% drop in the occurrence rate of shoulder disorders in a study of high-performance tennis players who performed "sleeper stretch" exercises on a daily basis (they show improvement in the ROM and also in IR of the shoulders which focused on muscles like the infraspinatus and teres minor muscles) [31].

Peck et al. reinforce this notion by recommending dynamic stretching before exercise for most athletes and suggesting static stretching after exercise if used at all. This approach helps counteract any potential reduction in performance caused by static stretching prior to the workout [32]. The importance of understanding how stretching impacts injury risk cannot be overlooked. McHugh and Cosgrave emphasize that dynamic stretching before physical activities may benefit certain injuries more than static stretching. Hence, incorporating dynamic stretching into warm-up routines can be a smart choice for injury prevention [33].

Herman and Smith's study evaluates the impact of a dynamic warm-up intervention compared to a static warm-up on various performance measures in college wrestlers. By engaging large muscle groups in dynamic movements before activity-specific skills, athletes experience enhanced preparedness and performance [34]. Moreover, McClure et al., in their study, indicate that the cross-body stretch, a form of dynamic stretching, may be more beneficial than no stretching at all for individuals with restricted shoulder medial rotation (ROM) [35]. Samukawa and colleagues in 2011 examined the impact of dynamic stretching on ankle dorsiflexion ROM and reported a significant increase following the dynamic stretching regimen [16]. Duncan and Woodfield, back in 2006, explored the influence of dynamic warm-up compared to static warm-up on vertical jump height in children. Their findings showed a noteworthy improvement in vertical jump height after dynamic warm-up [36]. Yamauchi et al.'s 2016 study focused on modified cross-body and sleeper stretches, concluding that both stretches were effective in enhancing shoulder IR and horizontal adduction ROM while reducing muscle stiffness in the shoulder [13]. Perrier and his team in 2009 delved into the comparison of static stretching, dynamic stretching, and countermovement jump (CMJ) height. Their research demonstrated that dynamic stretching led to significantly higher CMJ height compared to no stretching and static stretching [37].

Discussion

Dynamic stretching is a crucial tool in injury prevention and rehabilitation, particularly for athletes and individuals with shoulder problems. Ribeiro et al. in their research emphasize its therapeutic potential in improving quality of life and upper limb function for those with rotator cuff injuries [28]. Çelik in his study introduces cyclic stretching into sports and athletic performance, offering athletes a balanced approach that optimizes performance [29].

Christensen et al. in their research explore the life-changing potential of dynamic stretching for patients with non-repairable rotator cuff injuries, encouraging physiotherapists to incorporate exercise treatment into patient care [30]. Corpus et al. in their study highlight the importance of stretching exercises in sports, particularly for high-performance athletes, revealing substantial gains in total IR and reduced shoulder disorders [31]. Peck et al. recommendations suggest prioritizing dynamic stretching before exercise and judicious use of static stretching, providing athletes with an evidence-based approach to enhance performance while minimizing potential strength reduction [32]. Tawfik et al. investigate the effects of the sleeper stretch on shoulder ROM in collegiate athletes. The article discusses the technique of the sleeper stretch, its benefits in isolating the soft tissue restraints in the posterior shoulder, and its significant improvements in shoulder IR and horizontal adduction observed in collegiate baseball players [38]. Jo and Kim selected and analyzed nine out of 18 studies on stretching techniques. The study found that sleeper stretching and cross-body stretching techniques were applied for 30 seconds with three repetitions and found out that these techniques are very much beneficial [39]. From the above discussion, this review shows comparisons of specific stretching methods, their impact on flexibility, and potentially any associated benefits or risks.

The limitation of this review is the dearth of literature of higher levels of evidence articles like meta-analysis and systematic reviews.

## Conclusions

Prevention of rotator cuff injury should be improvised to decrease the risk of occurrence. Hence, our findings emphasize the significance of prioritizing prevention strategies to effectively reduce the risk of rotator cuff injuries. Our research strongly advocates the adoption of a structured approach to injury prevention and treatment. Incorporating dynamic stretching before engaging in exercises or workouts prepares the shoulder muscles for physical stress, reducing the likelihood of injury during physical activities. Additionally, incorporating static stretching after workouts aids in enhancing flexibility and promoting muscle recovery, further reducing the risk of future injuries. By integrating these preventive and treatment strategies, we can significantly enhance the overall shoulder health and athletic performance of individuals. By making a thoughtful selection and timing of stretching techniques, individuals can experience improved flexibility, strength, and injury prevention. For optimal results, dynamic stretching should be prioritized as a warm-up method before engaging in physical activities. Future research in this area should target on the long-term effects of these measures and explore additional interventions to strengthen the rotator cuff and surrounding structures. Ultimately, embracing a comprehensive approach to rotator cuff care will contribute to better musculoskeletal health and a higher quality of life for individuals across various age groups and physical activity levels.
